# Supplementary material for: Sis1 potentiates the stress response to protein aggregation and elevated temperature
Source: Nat Commun. 2020 Dec 8;11:6271. doi: 10.1038/s41467-020-20000-x (PMC7722728; doi:10.1038/s41467-020-20000-x)
Supplement: Supplementary file 1 — Supplementary Information [file 41467_2020_20000_MOESM1_ESM.pdf]

Supplementary information

Sis1 potentiates the stress response to protein aggregation and elevated temperature

Courtney L. Klaips[1,2], Michael H. M. Gropp[1], Mark S. Hipp[2,3] and F. Ulrich Hartl[1,*]

[1]Department of Cellular Biochemistry, Max Planck Institute of Biochemistry, Am Klopferspitz 18, 82152 Martinsried, Germany
[2]Department of Biomedical Sciences of Cells and Systems, University Medical Center Groningen, University of Groningen, Antonius Deusinglaan 1, 9713 AV Groningen, The Netherlands
[3]School of Medicine and Health Sciences, Carl von Ossietzky University Oldenburg, Oldenburg, Germany

*Correspondence and requests for materials should be addressed to F.U.H. (email: uhartl@biochem.mpg.de)

Supplementary information includes 7 figures and 7 tables.

Supplementary Figures

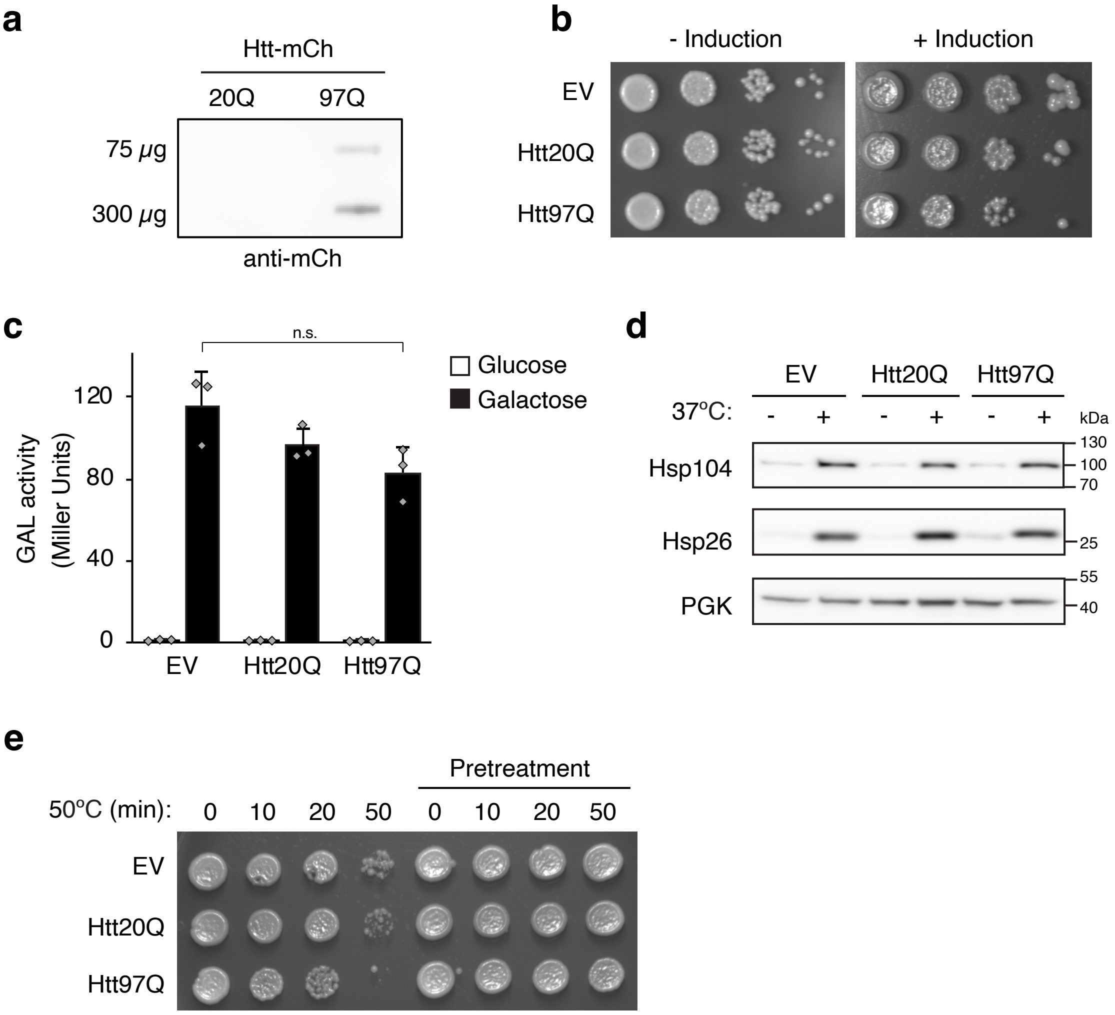

Supplementary Fig. 1 | Aggregation of polyQ expansion protein does not trigger a stress response in yeast. a, Htt97Q-mCh forms SDS resistant aggregates. Lysates from cells expressing Htt20Q-mCh or Htt97Q-mCh corresponding to 75 and 300 μg total protein were filtered through a 0.2 μm cellulose acetate membrane after treatment with 2% SDS. The membrane was then processed for immunodetection with anti-mCherry antibody. A representative result is shown from experiments performed in triplicate. **b,** Expression of Htt97Q in yeast is not overtly toxic. Yeast cells expressing Htt20Q or Htt97Q and empty vector (EV) control were serially diluted and plated on non-inducing (glucose) or inducing (galactose) media. **c,** Htt97Q aggregates have only a minor effect on β-galactosidase activity levels. β-Galactosidase activity was measured in cells expressing Htt20Q or Htt97Q and containing a LacZ reporter under the control of a galactose inducible promoter (GAL), grown in glucose or galactose containing media. Data represent mean + SD from 3 independent experiments. n.s., $p > 0.05$ by unpaired, two-sided t-test. **d,** The heat-induced cytosolic stress response remains active in polyQ expressing cells. Lysates from cells expressing Htt20Q, Htt97Q or empty vector (EV) control grown at 30°C and either maintained at 30°C or shifted to 37°C for 1 h were analyzed by SDS-PAGE and immunoblotting for Hsp104, Hsp26, or PGK. Representative results of 3 independent

experiments are shown. **e,** Mild heat treatment protects Htt97Q expressing cells against lethal heat exposure. Growth at 30ºC was analyzed in cells expressing Htt20Q, Htt97Q or empty vector (EV) control either grown at 30ºC before exposure to a lethal stress at 50ºC (left) or first exposed to a mild heat stress for 1 h at 37ºC, followed by 50ºC heat stress (right).

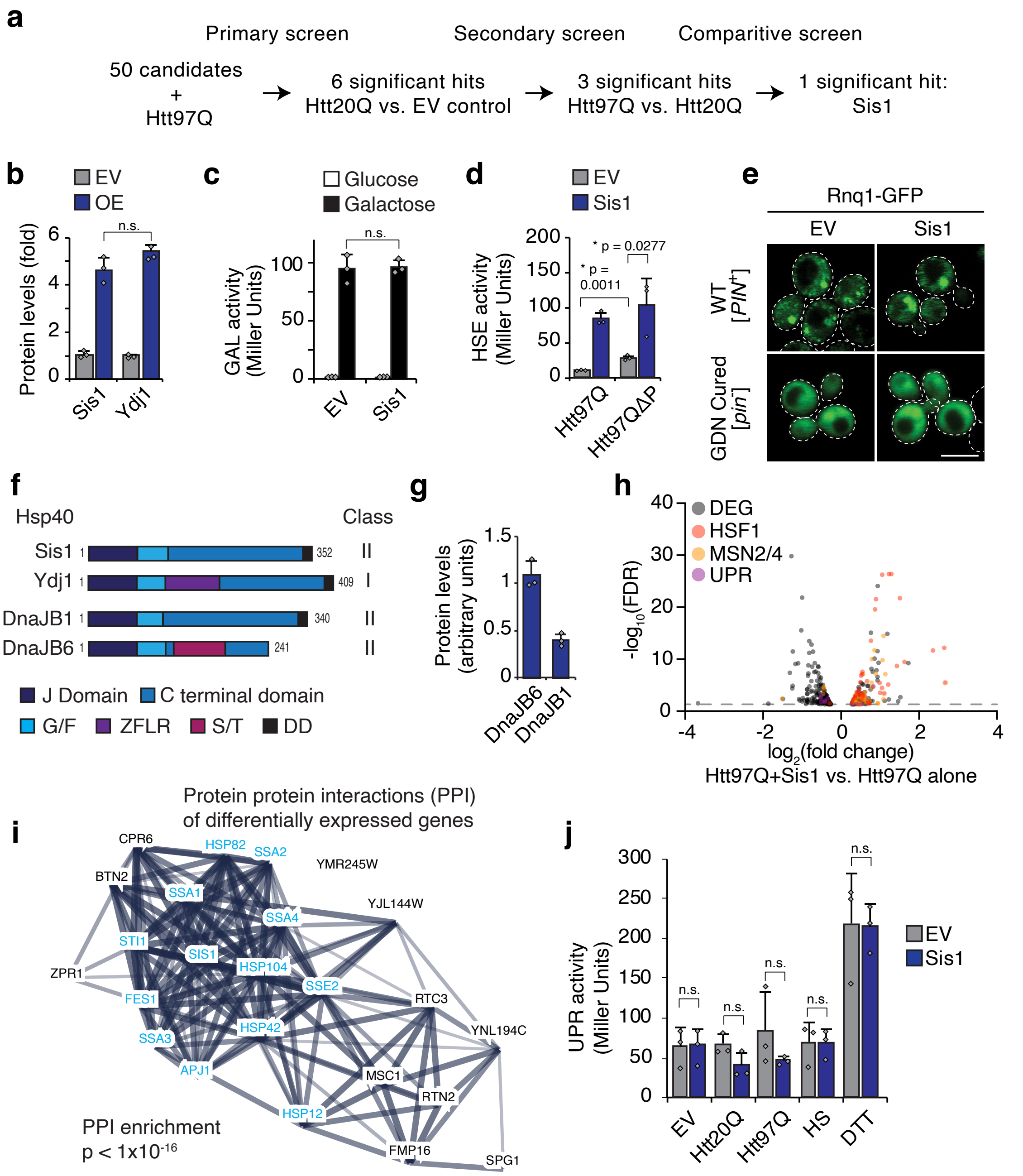

Supplementary Fig. 2 | Sis1 enables stress response activation by polyQ expansion protein. a, Schematic representation of chaperone screen strategy. In the primary screen, Htt97Q and each of 50 chaperones available in a 2µ library was expressed in cells containing P_{HSE}LacZ. The chaperones identified as enabling a HSR were next expressed with Htt20Q to select against those having general stress response effects. Finally, chaperones were identified that allowed HSR induction by Htt97Q that was significantly greater than induction by Htt20Q. See also Supplementary Table 1. **b,** Sis1 and Ydj1 overexpression levels. Lysates from cells expressing either an empty vector control, Sis1 or Ydj1 from a centromeric vector under the *GPD* promoter were analyzed by SDS-PAGE and immunoblotting for Sis1 or Ydj1 and PGK as a loading control, followed by densitometry. Data represent mean + SD from 3 independent experiments. n.s., $p > 0.05$ by unpaired, two-sided t-test. See also Supplementary Fig. 6a. **c,** A LacZ reporter containing a galactose-inducible promoter is not affected by

Sis1 overexpression. β-Galactosidase activity was measured in cells containing P_{GAL}LacZ with or without Sis1 overexpression after growth in glucose (repressive) or galactose (inducing). Data represent mean + SD from 3 independent experiments. n.s., $p > 0.05$ by unpaired, two-sided t-test. **d,** A toxic Htt exon-1 construct lacking the poly-proline region (Htt97QΔP) induces the stress response in a Sis1 dependent manner. β-Galactosidase activity was measured in cells expressing Htt97Q or Htt97QΔP with and without Sis1 overexpression. Data represent mean + SD from 3 independent experiments. *, p values are reported for unpaired, two-sided t-tests. **e,** The PIN prion status of cells is not affected by Sis1 overexpression. Confocal imaging of cells expressing Rnq1-GFP with or without Sis1 overexpression. Scale bar = 5µm. Experiments were performed in triplicate, representative images shown. **f,** Domain topology of selected J-domain proteins. General domain topology for Sis1, Ydj1, DnaJB1 and DnaJB6 is shown. G/F, glycine, phenylalanine rich region; ZFLR, zinc-finger like region; S/T, serine, threonine rich region; DD, dimerization domain. **g,** DnaJB6 and DnaJB1 overexpression levels. Lysates from cells expressing V5-tagged DnaJB6 or DnaJB1 from a centromeric vector under the *GPD* promoter were analyzed by SDS-PAGE and immunoblotting for V5 and PGK as a loading control, followed by densitometry. Data represent mean + SD from 3 independent experiments. **h,** Volcano plot of genes that were significantly differentially expressed (DEG) between cells expressing Htt97Q alone vs. Htt97Q with Sis1 overexpression are shown on a volcano plot. Dashed line = p adjusted 0.05. Common stress operons are marked (HSF1, red; MSN2/4, orange; UPR, purple). Due to scale, the exogenously overexpressed Sis1 is not shown ($\log_2$ fold change = 4.5406, $-\log_{10}$(FDR) = 97.7073). See also Supplementary Table 3. **i,** Protein-protein interaction map for genes enriched upon Sis1 expression with Htt97Q, as determined by mRNA sequencing. Differentially expressed genes enriched more than two-fold upon co-overexpression of Sis1 with Htt97Q compared to expression of Htt97Q alone were analyzed for protein-protein interactions (PPI). Line thickness represents relative number of identified interactions in the String database (string-db.org). Chaperone proteins and co-factors are indicated in blue. **j,** A LacZ reporter containing an unfolded protein response (UPR) inducible promoter is not induced by Htt97Q or Sis1 expression. β-Galactosidase activity was measured in cells containing P_{UPR}LacZ and Htt20Q, Htt97Q or empty vector (EV) control with or without Sis1 overexpression at normal temperature of 30°C after exposure to heat stress at 37°C for 1 h (HS), or after treatment with DTT (2 mM). Data represent mean + SD from 3 independent experiments. n.s., $p > 0.05$ by unpaired, two-sided t-test.

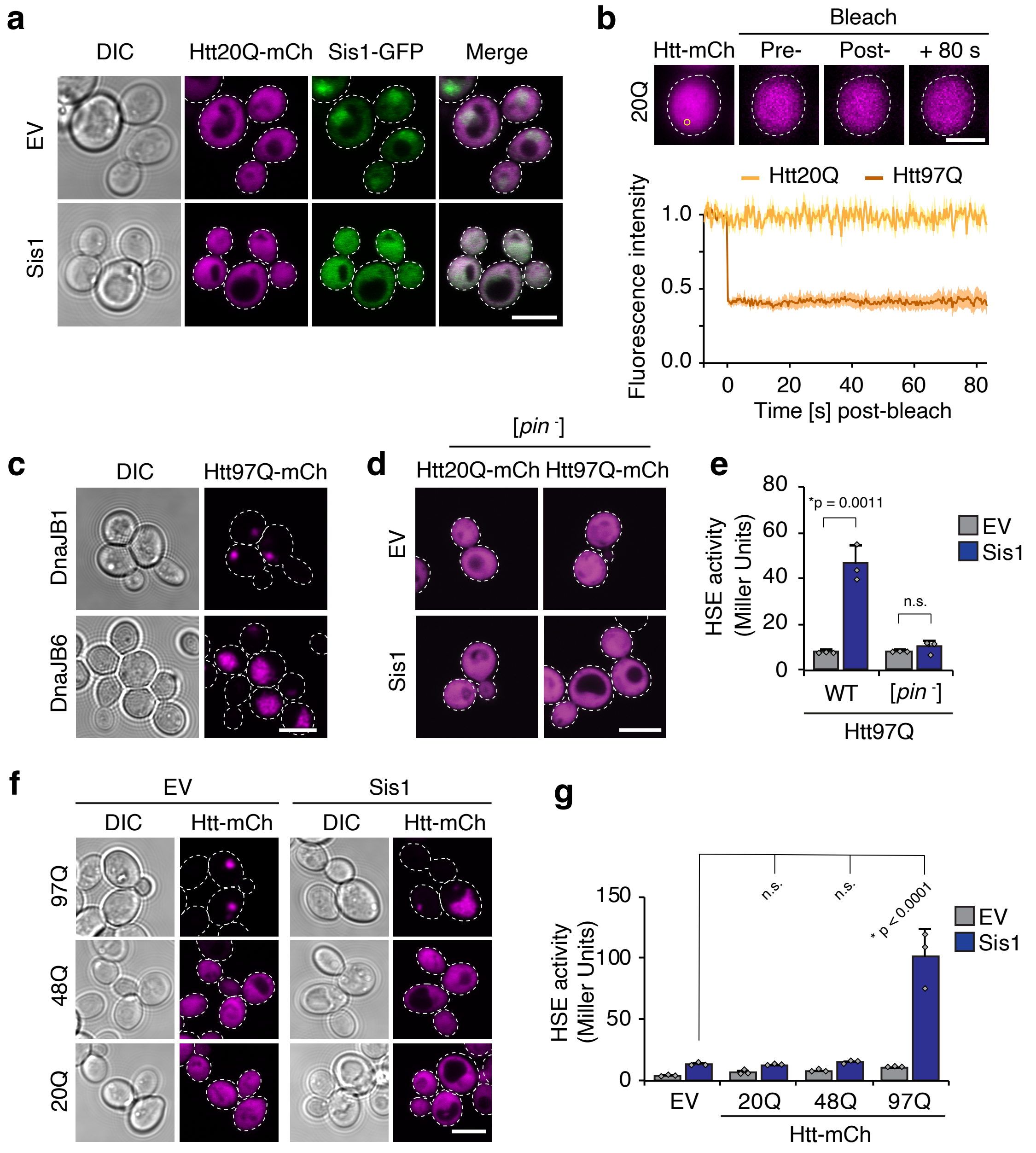

Supplementary Fig. 3 | Sis1 affects physico-chemical aggregate properties. a, Sis1 localizes to the cytosol and nucleus. Confocal images of cells containing endogenously tagged Sis1-GFP and expressing Htt20Q with or without Sis1 overexpression as in Fig. 2. Scale bar = 5 µm. Experiments were performed in triplicate, representative images shown. See also Fig. 3a. **b,** Soluble Htt20Q is highly mobile. Fluorescence recovery after photo bleaching (FRAP) experiments were performed on cells expressing soluble Htt20Q-mCherry. Representative images pre- and post-bleach are shown (top), scale bar = 2.5µm. Data represent mean (solid lines) ± SE (traces) of fluorescence intensity of at least 3 experimental replicates (bottom). Htt97Q is shown as a comparison (see also Fig. 3d). **c,** DnaJB6 affects Htt aggregates. Confocal microscopy of cells expressing Htt97Q with overexpression of DnaJB1 or DnaJB6. Scale bar = 5µm. Experiments were performed in triplicate, representative images shown. **d,** Htt97Q is diffusely distributed in [*pin*⁻] cells. Confocal microscopy was performed on [*pin*⁻] cells expressing Htt20Q or Htt97Q. Scale bar = 5µm.

Experiments were performed in triplicate, representative images shown. **e,** The stress response to Htt97Q is dependent on prion aggregates. β-Galactosidase activity was measured in WT [*PIN*$^+$] or [*pin*$^-$] cells containing P_{HSE}LacZ and Htt97Q with or without Sis1 overexpression. EV, empty vector. Data represent mean + SD from 3 independent experiments. *, p values were calculated by unpaired, two-sided t-test. n.s., not statistically significant. **f,** Medium length polyQ-expanded Htt (Htt48Q) does not form aggregates. Confocal microscopy was performed on cells expressing Htt20Q, Htt48Q or Htt97Q with or without Sis1 overexpression. EV, empty vector. Scale bar = 5μm. Experiments were performed in triplicate, representative images shown. **g,** Sis1 dependent HSR induction is dependent on polyQ length. P_{HSE}LacZ activity was measured in cells expressing Htt20Q, Htt48Q or Htt97Q with or without Sis1 overexpression. EV, empty vector. Data represent mean + SD of 3 independent experiments. *, p values were determined by Dunnett's multiple comparisons t-test to EV control with Sis1. n.s., not statistically significant.

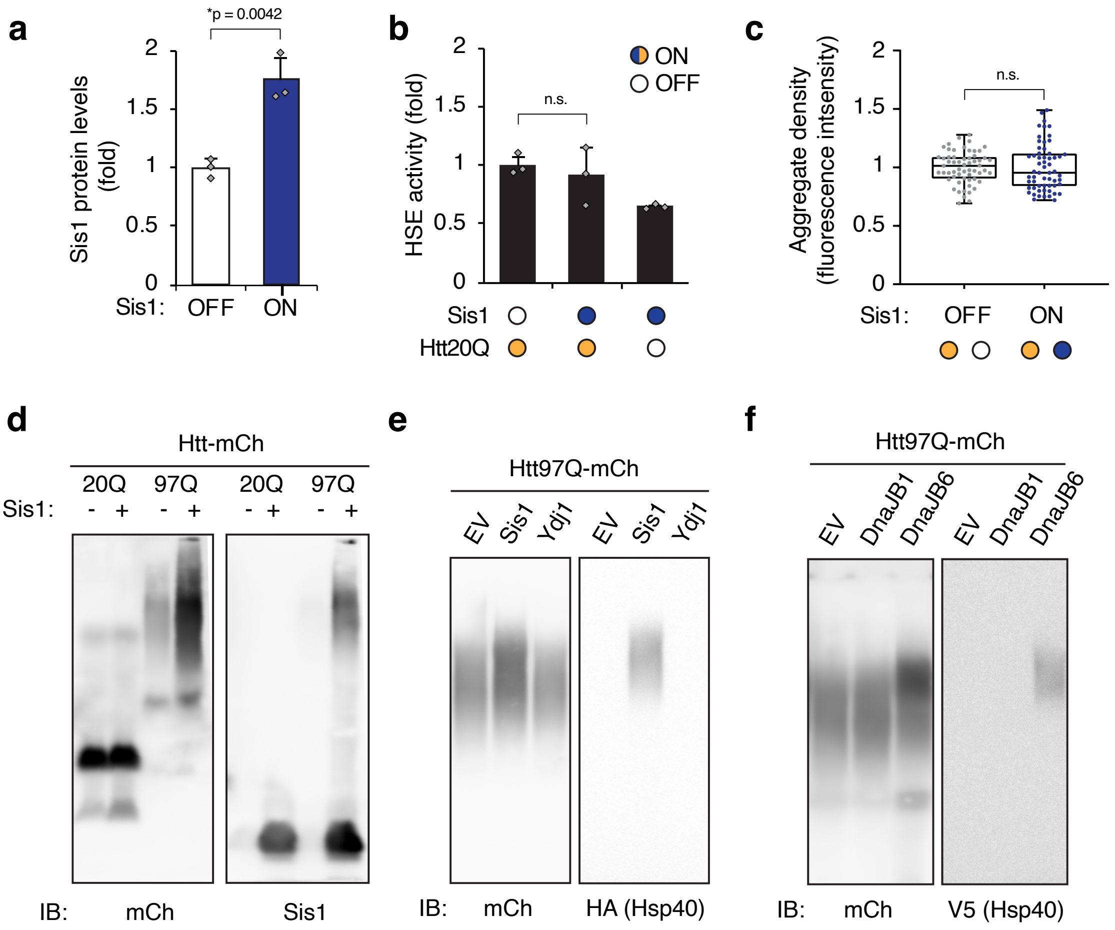

Supplementary Fig. 4 | Sis1 acts on soluble polyQ oligomers. a, Levels of Sis1 upon transient overexpression. Lysates from cells with or without transient Sis1 overexpression were analyzed by SDS-PAGE and immunoblotting for Sis1 and PGK as a loading control. Data represent mean + SD from 3 independent experiments. *, p value was determined using unpaired, two-sided t-test. **b,** Htt20Q expression does not induce a stress response with or without transient Sis1 expression. P_{HSE}LacZ activities were measured in cells treated as in Fig. 4a. Htt20Q was expressed in cells for ~18 h (orange dot). Htt20Q expression was then continued for 3 h without or with Sis1 overexpression (blue and orange dot). In a third reaction, Htt20Q expression was stopped by the addition of glucose and Sis1 was overexpressed for 3 h (blue dot). P_{HSE}LacZ activities were measured. Experiments were performed in triplicate. Data represent mean + SD from 3 independent experiments. n.s., p > 0.05 by unpaired, two-sided t-test. See also Fig. 4b. **c,** Preformed Htt97Q aggregates are of similar density with and without transient overexpression of Sis1. Confocal microscopy was performed on cells expressing Htt97Q for 24 h (orange dot) without Sis1 overexpression (Sis1 OFF; empty dot) or with Sis1 overexpression for the final 3 h (Sis1 ON; blue dot). Average fluorescence intensity of aggregates was measured (see Methods for details). Box plots represent median and 25th and 75th percentile, and whiskers minimal and maximal values. n = 59 cells. n.s., p > 0.05 by unpaired, two-sided t-test. See also Fig. 4c. **d,** Sis1 mediated polyQ oligomer formation is specific to Htt97Q. Lysates from cells expressing Htt20Q or Htt97Q with or without Sis1

overexpression were analyzed by SDD-AGE and immunoblotted for mCherry (Htt) or Sis1 as in Fig. 4d. Representative results of 3 independent experiments are shown. **e,** Ydj1 does not bind to soluble Htt97Q oligomers. Lysates from cells expressing Htt97Q with or without HA- tagged Sis1 or Ydj1 overexpression were analyzed by SDD-AGE and immunoblotting for mCherry (Htt97Q) or HA (Sis1, Ydj1) as in (d). EV, empty vector. **f,** DnaJB6 binds to soluble Htt97Q oligomers. Lysates from cells expressing Htt97Q with overexpression of DnaJB1 or DnaJB6 were analyzed by SDDAGE. Immunoblotting was performed for mCherry (Htt97Q) and the V5 epitope (DnaJB1 and DnaJB6). EV, empty vector. Representative blots of 3 independent experiments are shown.

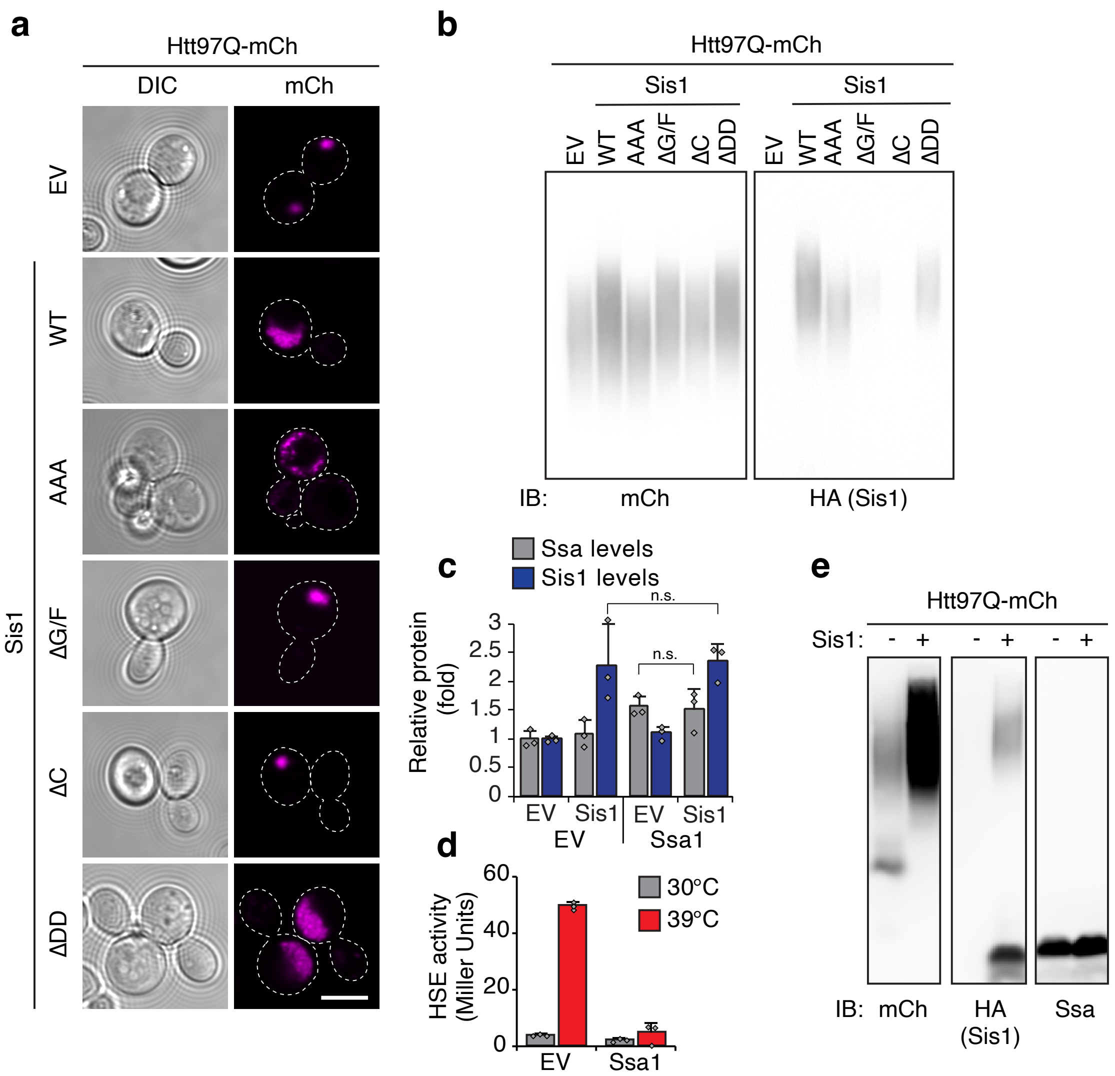

Supplementary Fig. 5 | Sis1 recruits Hsp70 (Ssa1/2) to polyQ expanded Htt. a, Formation of cloud-like polyQ condensates corresponds to stress response induction. Confocal imaging was performed on cells expressing Htt97Q and overexpression of WT Sis1 and Sis1 mutants (see Fig. 5a, b). EV, empty vector. Scale bar = 5µm. Experiments were performed in triplicate, representative images shown. **b,** Sis1 mutants Sis1ΔG/F and Sis1ΔC are defective in binding Htt97Q oligomers. Lysates from cells expressing Htt97Q and HA-tagged Sis1 proteins were analyzed by SDD-AGE and immunoblotting for mCherry (Htt97Q) and HA (Sis1). Representative blots of 3 independent experiments are shown. **c,** Ssa1 overexpression levels. Cells overexpressing Sis1 and Ssa1 either alone or in combination were analyzed by SDS-PAGE and immunoblotting for Ssa1/2, Sis1, and PGK. Note that due to a high level of homology, the Ssa antibody used recognizes both Ssa1 and Ssa2. Data represent mean + SD of three experiments. n.s., $p > 0.05$ by unpaired, two-sided t-test. **d,** Ssa1 overexpression blocks the heat induced stress response. P_{HSE}LacZ activity was measured in cells with or without Ssa1 overexpression grown at 30ºC with or without a 1 h treatment at 39ºC. Data represent mean + SD from 3 independent experiments. **e,** Interaction of Htt97Q with Ssa1/2 is less stable than with Sis1. Lysates from cells expressing Htt97Q with or without HA-Sis1 overexpression were analyzed by SDD-AGE as in (b). Immunoblotting was performed for mCherry (mCh, Htt97Q), HA (Sis1), or Ssa1/2. Representative blots from 3 experiments are shown.

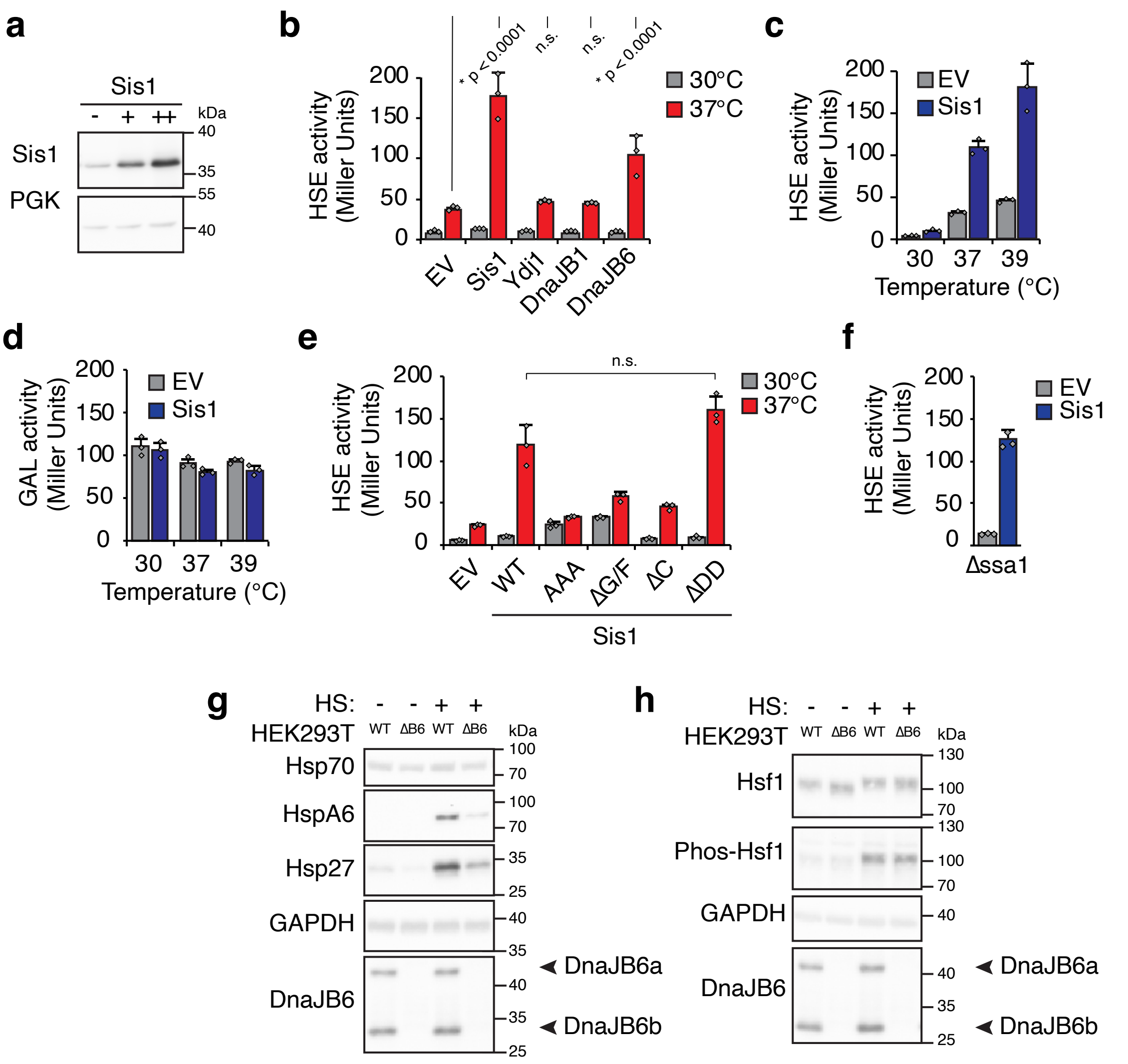

Supplementary Fig. 6 | Sis1 overexpression enhances the cytosolic stress response. a, Sis1 overexpression under different promoters. Lysates from cells expressing Sis1 at endogenous (EV, -), mildly elevated (P_{CYC}Sis1, +) or highly elevated (P_{GPD}Sis1, ++) levels were analyzed by SDS-PAGE and immunoblotting for Sis1 and PGK as a loading control as in Fig. S2B. Representative blots from 3 independent experiments are shown. **b,** DnaJB6 enhances the heat induced stress response. β-galactosidase activity was measured in yeast cells expressing various Hsp40s grown at 30°C and either maintained at 30°C or exposed to 37ºC for 1 h. EV, empty vector. Data represent mean + SD from 3 independent experiments. *, p values were calculated by Dunnett's multiple comparisons t-test to EV control at 37ºC. n.s., not statistically significant. **c,** Sis1 enhances the stress response at different temperatures. β-galactosidase activity was measured in cells with or without Sis1 overexpression grown at 30ºC and either maintained at 30ºC or shifted to 37ºC or 39ºC for 1 h. Data represent mean + SD from 3 independent experiments. **d,** Increased temperature does not affect reporter fidelity with or without Sis1 overexpression. β-Galactosidase activity was measured in cells containing the galactose-inducible P_{GAL}LacZ reporter, grown in inducing media (galactose) and treated at different temperatures as in (c) with or without Sis1 overexpression. Data represent mean + SD from 3 independent experiments. **e,** Mutational analysis of Sis1 function in enhancing heat induced stress activation. P_{HSE}LacZ activity was measured in cells overexpressing various Sis1 mutant

proteins (see Fig. 5a, b) and exposed to 37°C heat stress as in (b). Data represent mean + SD from 3 independent experiments. n.s., $p > 0.05$ by unpaired, two-sided t-test. **f,** Sis1 overexpression causes a stress response in Δ*ssa1* cells at normal growth temperature. β-Galactosidase activity was measured in cells lacking *SSA1* with or without Sis1 overexpression at 30°C. Data represent mean + SD from 3 independent experiments. **g,** DnaJB6 is required for efficient heat induced stress response in mammalian cell culture. Lysates from WT HEK293T cells or cells deleted for DnaJB6 (ΔB6) grown at 37°C without or with a 1 h treatment at 43°C and recovery (heat stress, HS) were analyzed by SDS-PAGE and immunoblotting for the indicated proteins. Blots are representative of three independent experiments. **h,** DnaJB6 is not required for efficient phosphorylation of HSF1 during heat stress. Lysates from WT HEK293T cells or cells deleted for DnaJB6 (ΔB6) grown at 37°C without or with a 1 h treatment at 43°C treatment (HS) were analyzed by SDS-PAGE and immunoblotting for the indicated proteins. Blots are representative of three independent experiments.

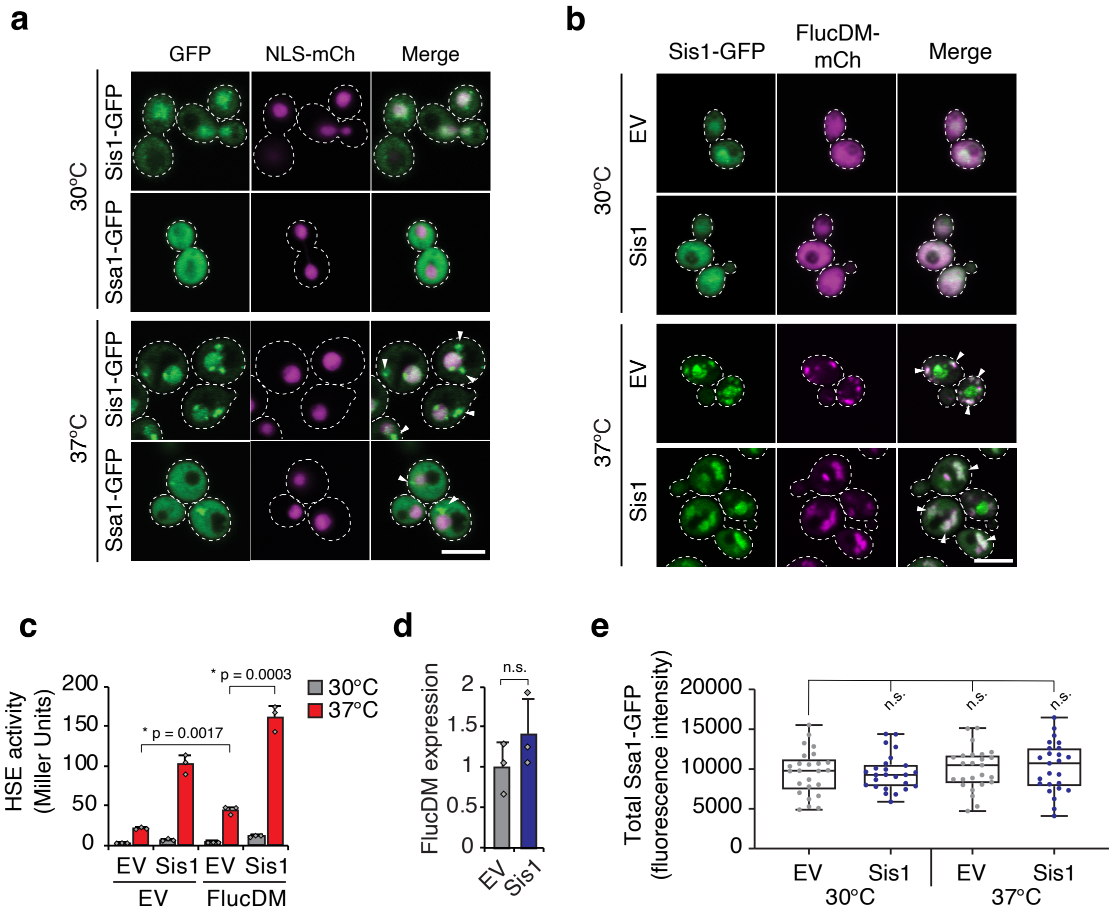

Supplementary Fig. 7 | Sis1 recruits Ssa1 to heat-induced protein aggregates. a, Confocal microscopy was performed on cells containing an endogenously tagged *SIS1-GFP* or a copy of *SSA1-GFP* under the *ADH* promoter and expressing nuclear targeted mCherry grown at 30°C. Cells were either maintained at 30°C or incubated at 37°C for 1 h. White arrows indicate cytosolic foci. Scale bars = 5μm. Experiments were performed in triplicate, representative images shown. **b,** Co-localization of Sis1 with FlucDM aggregates. Confocal microscopy was performed on cells containing an endogenously tagged *SIS1-GFP* and expressing FlucDM with or without Sis1 co-overexpression grown at 30°C and either maintained at 30°C or incubated at 37°C for 1 h. White arrows indicate co-localized aggregates. Scale bars = 5μm. Experiments were performed in triplicate, representative images shown. **c,** FlucDM induced stress response is enhanced by Sis1. P_{HSE}LacZ reporter cells expressing either EV or FlucDM-mCh with or without Sis1 overexpression were subjected to heat stress at 37°C for 1 h before measurement of β-galactosidase activity. Data represent mean + SD from 3 independent experiments. *, p values represent the results from unpaired, two-sided t-tests. **d,** FlucDM-mCh expression was measured in strains with or without Sis1 overexpression. Data represent mean + SD from 3 experiments. n.s., p > 0.05 by unpaired, two-sided t-test. **e,** Total Ssa1-GFP levels were measured by fluorescence in *SSA1-GFP* strains expressing FlucDM-mCh with or without Sis1 overexpression grown at 30°C and either maintained at 30°C or incubated at 37°C for 1 h. Box plots represent median and 25th and 75th percentile, and whiskers minimal and maximal values. n = 25 cells. n.s., p > 0.05 by Dunnett's multiple comparisons t-test to EV control cells at 30°C. See also Fig. 6d.

Supplementary Tables

Supplementary Table 1│ Chaperone screen for Htt97Q mediated induction of the stress response. P_{HSE}LacZ activity (HSE activity) was measured in cells expressing Htt97Q and each of the listed chaperone proteins from a 2-micron, multi-copy, galactose-inducible library. Red, high; blue, low HSE activity. Significant hits were subjected to further screening in triplicate with Htt20Q and Htt97Q. Secondary hits identified as Htt97Q specific (p >0.05 for Htt20Q vs. empty vector (EV) and p <0.05 for Htt97Q vs. Htt20Q by unpaired, two-sided t-test) are marked in bold. See also Fig. 2a and Supplementary Fig. 2a.

Primary Screen

Locus	Name	HSE Activity	Type
YBR227C	MCX1	13	AAA+
YPL235W	RVB2	23	AAA+
YDR212W	CCT1	21	CCT
YDL143W	CCT4	25	CCT
YDR188W	CCT6	32	CCT
YJL111W	CCT7	27	CCT
YJL008C	CCT8	14	CCT
YOR020C	HSP10	13	Hsp10
YER048C	CAJ1	17	Hsp40
YIR004W	DJP1	28	Hsp40
YFR041C	ERJ5	28	Hsp40
YJL073W	JEM1	30	Hsp40
YNL227C	JJJ1	29	Hsp40
YNL328C	MDJ2	21	Hsp40
YNL007C	SIS1	62	Hsp40
YLR008C	TIM14	16	Hsp40
YLR090W	XDJ1	57	Hsp40
YNL064C	YDJ1	49	Hsp40
YGR285C	ZUO1	40	Hsp40
YLR259C	HSP60	12	Hsp60
YJL034W	KAR2	31	Hsp70
YKL073W	LHS1	23	Hsp70
YAL005C	SSA1	19	Hsp70
YLL024C	SSA2	20	Hsp70
YBL075C	SSA3	21	Hsp70
YER103W	SSA4	15	Hsp70
YNL209W	SSB2	38	Hsp70
YLR369W	SSC2	14	Hsp70
YEL030W	SSC3	14	Hsp70
YPL106C	SSE1	70	Hsp70
YBR169C	SSE2	53	Hsp70
YHR064C	SSZ1	54	Hsp70
YOL031C	SIL1	35	Hsp70 cofactors
YIL016W	SNL1	26	Hsp70 cofactors
YMR186W	HSC82	41	Hsp90
YPL240C	HSP82	35	Hsp90
YDR214W	AHA1	29	Hsp90 cofactors
YDR168W	CDC37	39	Hsp90 cofactors
YJR032W	CPR7	31	Hsp90 cofactors
YNL281W	HCH1	27	Hsp90 cofactors
YHR034C	PIH1	11	Hsp90 cofactors
YOR027W	STI1	32	Hsp90 cofactors
YCR060W	TAH1	18	Hsp90 cofactors
YEL003W	GIM4	16	Prefoldin
YFL014W	HSP12	24	Small heat shock
YDR533C	HSP31	15	Small heat shock
YPL280W	HSP32	20	Small heat shock
YOR391C	HSP33	17	Small heat shock
YMR322C	HSP34	17	Small heat shock
YDR171W	HSP42	23	Small heat shock
	Avg	28	
	SD	14	

Secondary Screen

Htt20Q	Avg	SD	p (vs. EV)	p (97 vs. 20)
EV	12.826576	2.1846541	-	0.9982
SSE1	60.532422	7.810914	0.0005	0.5111
SIS1	24.100524	8.9442409	**0.1012**	**0.0413**
XDJ1	28.202575	12.104521	**0.0963**	0.1935
SSZ1	36.543376	3.5865615	0.0006	**0.0485**
SSE2	37.167786	4.2486281	0.0009	0.2203
YDJ1	57.066307	32.236623	**0.0767**	0.3478

Htt97Q	Avg	SD	p (vs. EV)
EV	12.841012	9.9847483	-
SSE1	57.158045	2.1906806	0.0017
SIS1	39.740551	1.8467804	0.0101
XDJ1	42.686363	10.566526	0.0237
SSZ1	52.343609	9.0704405	0.0071
SSE2	46.181645	9.8836444	0.0147
YDJ1	36.908868	6.346612	0.0244

Supplementary Table 2│ GO term enrichment over control. mRNA sequencing was performed on cells expressing empty vector (EV) control, Sis1, Htt97Q, and Sis1 with Htt97Q. The top GO biological processes are listed for the differentially upregulated genes for each condition compared to the EV control. See also Fig. 2c. p values were calculated using the GOseq R package, which corrects for gene length bias. Black text indicates significant terms (p <0.05), gray text represents not significant terms (p >0.05).

Sis1 vs. EV

GO_accession	p value	Description
GO:0006635	0.4402	fatty acid beta-oxidation
GO:0019395	0.4402	fatty acid oxidation
GO:0034440	0.4402	lipid oxidation
GO:0009062	0.64612	fatty acid catabolic process
GO:0044282	0.70669	small molecule catabolic process
GO:0044767	0.70669	single-organism developmental process
GO:0032502	0.70669	developmental process
GO:0072329	0.70669	monocarboxylic acid catabolic process
GO:0033540	0.70669	fatty acid beta-oxidation using acyl-CoA oxidase
GO:0003006	0.70669	developmental process involved in reproduction
GO:1901135	0.76527	carbohydrate derivative metabolic process
GO:0044242	0.76527	cellular lipid catabolic process
GO:0030258	0.76527	lipid modification
GO:0044712	0.94661	single-organism catabolic process
GO:0016042	1	lipid catabolic process
GO:0009653	1	anatomical structure morphogenesis
GO:0048856	1	anatomical structure development
GO:0055114	1	oxidation-reduction process
GO:0070843	1	misfolded protein transport
GO:0019953	1	sexual reproduction

Htt97Q vs. EV

GO_accession	p value	Description
GO:0042254	9.51E-20	ribosome biogenesis
GO:0034470	8.98E-18	ncRNA processing
GO:0022613	3.39E-17	ribonucleoprotein complex biogenesis
GO:0006364	3.39E-17	rRNA processing
GO:0016072	4.07E-16	rRNA metabolic process
GO:0034660	1.24E-15	ncRNA metabolic process
GO:0042274	3.37E-12	ribosomal small subunit biogenesis
GO:0000462	6.01E-12	maturation of SSU-rRNA from tricistronic rRNA transcript (SSU-rRNA, 5.8S rRNA, LSU-rRNA)
GO:0030490	1.71E-11	maturation of SSU-rRNA
GO:0042273	3.91E-10	ribosomal large subunit biogenesis
GO:0006396	6.49E-10	RNA processing
GO:0000460	1.79E-07	maturation of 5.8S rRNA
GO:0000466	1.79E-07	maturation of 5.8S rRNA from tricistronic rRNA transcript (SSU-rRNA, 5.8S rRNA, LSU-rRNA)
GO:0010467	3.80E-06	gene expression
GO:0000967	1.68E-05	rRNA 5'-end processing
GO:0034471	1.68E-05	ncRNA 5'-end processing
GO:0000469	1.81E-05	cleavage involved in rRNA processing
GO:0000966	3.32E-05	RNA 5'-end processing
GO:0000480	5.21E-05	endonucleolytic cleavage in 5'-ETS of tricistronic rRNA transcript (SSU-rRNA, 5.8S rRNA, LSU-rRNA)
GO:0044085	5.21E-05	cellular component biogenesis

Htt97Q+Sis1 vs. EV

GO_accession	p value	Description
GO:0006457	3.53E-13	protein folding
GO:0042026	3.75E-12	protein refolding
GO:0043248	2.65E-05	proteasome assembly
GO:0009266	0.000195	response to temperature stimulus
GO:0006950	0.000345	response to stress
GO:0009408	0.000345	response to heat
GO:0009628	0.001923	response to abiotic stimulus
GO:0043462	0.001982	regulation of ATPase activity
GO:0006200	0.003947	obsolete ATP catabolic process
GO:0033554	0.005941	cellular response to stress
GO:0006515	0.005941	misfolded or incompletely synthesized protein catabolic process
GO:0006616	0.006065	SRP-dependent cotranslational protein targeting to membrane, translocation
GO:0032781	0.006065	positive regulation of ATPase activity
GO:0006458	0.006072	'de novo' protein folding
GO:0034605	0.006092	cellular response to heat
GO:0042274	0.007524	ribosomal small subunit biogenesis
GO:0051603	0.01119	proteolysis involved in cellular protein catabolic process
GO:0044257	0.013268	cellular protein catabolic process
GO:0030490	0.017543	maturation of SSU-rRNA
GO:0045047	0.020527	protein targeting to ER

Supplementary Table 3│ mRNA enrichment with Htt97Q and Sis1. mRNA sequencing was performed on cells expressing Htt97Q without or with Sis1 overexpression. Genes enriched with Sis1 overexpression are listed. p values were determined using the DESeq R package and adjusted using the Benjamini and Hochberg method. See also Fig. 2d and Supplementary Fig. 2h, i.

ID	log2FoldChange	p value	Gene name
YNL007C	4.541	1.962E-98	SIS1
YER103W	2.675	3.405E-06	SSA4
YGR142W	2.645	6.607E-13	BTN2
YPL240C	2.358	2.010E-12	HSP82
YDR070C	1.727	5.483E-10	FMP16
YGR211W	1.631	3.211E-10	ZPR1
YNL194C	1.522	2.107E-03	-
YLL024C	1.508	1.792E-22	SSA2
YGR236C	1.465	1.274E-02	SPG1
YBR101C	1.434	2.113E-09	FES1
YOR027W	1.293	4.298E-27	STI1
YAL005C	1.228	4.298E-27	SSA1
YBL075C	1.224	5.748E-03	SSA3
YDR171W	1.212	7.672E-06	HSP42
YMR245W	1.190	1.294E-15	-
YML128C	1.134	7.825E-08	MSC1
YJL144W	1.118	1.003E-02	-
YBR169C	1.104	3.855E-04	SSE2
YDL204W	1.090	4.790E-03	RTN2
YFL014W	1.089	3.170E-15	HSP12
YHR087W	1.063	1.570E-10	RTC3
YLR216C	1.054	5.496E-27	CPR6
YLL026W	1.050	3.231E-05	HSP104
YNL077W	1.001	2.899E-04	APJ1
YDR155C	0.988	3.554E-06	CPR1
YMR169C	0.963	4.818E-10	ALD3
YMR175W	0.946	3.091E-02	SIP18
YEL021W	0.941	8.429E-25	URA3
YDL222C	0.940	3.161E-02	FMP45
YCL048W-A	0.936	1.174E-06	-
YKR106W	0.935	2.350E-07	GEX2
YNL195C	0.934	2.800E-12	-
YGR248W	0.907	1.260E-08	SOL4
YMR186W	0.897	2.713E-22	HSC82
YDL085W	0.881	5.379E-08	NDE2
YNL064C	0.874	2.688E-20	YDJ1
YLR109W	0.860	1.922E-12	AHP1
YMR090W	0.859	7.478E-11	-
YLR164W	0.828	1.735E-05	SHH4
YBR285W	0.810	2.583E-08	-
YLR178C	0.808	2.199E-05	TFS1
YMR174C	0.805	1.003E-02	PAI3
YOR173W	0.796	2.825E-05	DCS2
YGR043C	0.792	9.285E-11	NQM1
YMR250W	0.783	3.194E-06	GAD1

YOR161C	0.778	7.294E-09	PNS1
YDR214W	0.769	2.803E-14	AHA1
YOR007C	0.766	8.386E-16	SGT2
YOL032W	0.758	6.680E-05	OPI10
YNL006W	0.757	1.003E-02	LST8
YIL136W	0.734	1.058E-02	OM45
YDR258C	0.734	2.967E-02	HSP78
YKL065W-A	0.716	2.743E-02	-
YJL042W	0.704	3.262E-03	MHP1
YNR069C	0.702	2.779E-02	BSC5
YGR201C	0.697	1.370E-05	-
YCR012W	0.691	1.289E-02	PGK1
tN(GUU)P	0.687	2.642E-02	-
YDL024C	0.682	3.129E-02	DIA3
YJL163C	0.678	1.358E-05	-
YCL050C	0.667	1.643E-06	APA1
YIL101C	0.659	1.586E-02	XBP1
YHR054C	0.636	4.453E-02	-
YGL184C	0.618	1.970E-04	STR3
YPL123C	0.615	2.169E-03	RNY1
YER121W	0.612	3.241E-02	-
YPL124W	0.611	7.119E-03	SPC29
YFL016C	0.607	1.187E-03	MDJ1
YJR046W	0.601	2.243E-04	TAH11
YEL011W	0.600	6.063E-03	GLC3
YPL186C	0.579	2.587E-03	UIP4
YPL151C	0.569	7.330E-06	PRP46
YOR374W	0.569	2.087E-02	ALD4
YPR184W	0.567	8.357E-08	GDB1
YPR149W	0.563	7.373E-04	NCE102
YPR154W	0.556	7.684E-04	PIN3
YPL106C	0.547	2.644E-08	SSE1
YAL061W	0.546	2.734E-03	BDH2
YOL157C	0.544	3.795E-02	IMA2
YGL104C	0.544	6.692E-04	VPS73
YPL239W	0.543	6.834E-06	YAR1
YJL170C	0.541	1.933E-02	ASG7
YLR259C	0.541	6.935E-08	HSP60
YGR256W	0.540	5.114E-03	GND2
YDR089W	0.535	4.213E-02	-
YJL034W	0.533	4.039E-04	KAR2
YPL170W	0.531	3.902E-03	DAP1
YOL081W	0.529	3.945E-03	IRA2
YHR171W	0.521	5.956E-03	ATG7
YLR151C	0.519	4.097E-04	PCD1
YMR105C	0.518	3.418E-03	PGM2
YNL214W	0.516	1.320E-02	PEX17
YHR049W	0.516	3.554E-06	FSH1
YJL164C	0.512	4.321E-04	TPK1
YHR174W	0.512	1.465E-07	ENO2
YOR114W	0.502	1.752E-02	-
YER091C	0.498	5.742E-04	MET6
YOR020C	0.493	2.797E-06	HSP10

YIL119C	0.493	1.098E-02	RPI1
YBR157C	0.491	1.244E-02	ICS2
YLR082C	0.487	4.359E-03	SRL2
YPR026W	0.486	3.091E-02	ATH1
YBL039W-B	0.483	2.743E-02	-
YIR038C	0.476	3.877E-03	GTT1
YER096W	0.472	4.458E-02	SHC1
YFR053C	0.469	4.620E-02	HXK1
YNL281W	0.469	5.580E-05	HCH1
YHR104W	0.467	4.328E-02	GRE3
YOL151W	0.464	2.150E-02	GRE2
YLR327C	0.464	8.505E-04	TMA10
YPR160W	0.461	8.397E-04	GPH1
YNL300W	0.458	5.449E-05	TOS6
YGR284C	0.456	1.677E-04	ERV29
YDR151C	0.453	5.023E-04	CTH1
YKL035W	0.452	2.486E-05	UGP1
YGR008C	0.450	3.220E-02	STF2
YAL008W	0.448	3.346E-04	FUN14
YMR161W	0.440	9.808E-04	HLJ1
YIL139C	0.439	3.129E-02	REV7
YOL002C	0.432	2.350E-04	IZH2
YKL026C	0.427	3.705E-02	GPX1
YOR185C	0.427	1.428E-03	GSP2
YIL158W	0.426	6.242E-03	AIM20
YLL055W	0.426	1.393E-02	YCT1
YNL173C	0.426	3.552E-04	MDG1
YHR061C	0.424	3.084E-02	GIC1
YER142C	0.421	8.947E-03	MAG1
YBR026C	0.417	4.321E-04	ETR1
YJR047C	0.412	2.368E-02	ANB1
YMR196W	0.412	3.941E-02	-
YHR159W	0.411	3.593E-02	TDA11
YIR037W	0.410	3.952E-03	HYR1
YOR386W	0.410	3.112E-02	PHR1
YCL055W	0.409	2.180E-02	KAR4
YLR183C	0.406	1.751E-02	TOS4
YIL111W	0.406	1.700E-03	COX5B
YBR241C	0.405	5.956E-03	-
YGL038C	0.405	1.519E-02	OCH1
YBR067C	0.404	1.935E-04	TIP1
YKL042W	0.404	4.436E-02	SPC42
YHR096C	0.399	1.365E-02	HXT5
YPR175W	0.399	3.848E-02	DPB2
YOL082W	0.396	5.956E-03	ATG19
YGL208W	0.396	2.645E-02	SIP2
YDL019C	0.395	2.510E-02	OSH2
YPL004C	0.394	4.047E-04	LSP1
YOR215C	0.392	4.866E-03	AIM41
YDR453C	0.390	3.875E-02	TSA2
YAR015W	0.389	3.583E-02	ADE1
YNL289W	0.388	1.711E-03	PCL1
YNL098C	0.387	3.129E-02	RAS2

YHL030W	0.386	1.043E-02	ECM29
YPL096W	0.384	2.433E-02	PNG1
YKR076W	0.383	6.689E-03	ECM4
YOL096C	0.383	1.787E-02	COQ3
YBR139W	0.382	2.722E-03	-
YLR219W	0.380	4.039E-03	MSC3
YLR303W	0.379	1.235E-03	MET17
YFL029C	0.379	2.201E-02	CAK1
YGL093W	0.377	2.196E-02	SPC105
YBR231C	0.375	1.757E-02	SWC5
YDR330W	0.373	8.353E-03	UBX5
YER052C	0.373	1.794E-02	HOM3
YMR199W	0.370	2.695E-03	CLN1
YNL200C	0.368	4.007E-03	-
YDL197C	0.366	1.995E-02	ASF2
YOL153C	0.363	1.528E-02	-
YOL101C	0.358	2.857E-02	IZH4
YFR003C	0.357	4.557E-02	YPI1
YGR192C	0.356	1.140E-03	TDH3
YBR082C	0.352	2.335E-03	UBC4
YER079W	0.351	1.586E-02	-
YLR356W	0.351	1.113E-02	ATG33
YER001W	0.351	4.028E-03	MNN1
YAR007C	0.348	2.329E-02	RFA1
YBL064C	0.346	1.647E-02	PRX1
YIL042C	0.345	2.461E-02	PKP1
YLR324W	0.343	1.852E-02	PEX30
YDR399W	0.343	6.849E-03	HPT1
YOR228C	0.342	3.241E-02	MCP1
YMR031C	0.337	6.045E-03	EIS1
YGR086C	0.336	4.169E-03	PIL1
YOR020W-A	0.332	4.197E-02	-
YOL038W	0.328	9.527E-03	PRE6
YMR006C	0.325	1.431E-02	PLB2
YJL001W	0.324	1.003E-02	PRE3
YMR251W-A	0.321	5.177E-03	HOR7
YBR126C	0.320	2.087E-02	TPS1
YPL203W	0.318	2.874E-02	TPK2
YGL037C	0.318	7.268E-03	PNC1
YDL144C	0.318	3.965E-02	-
YBR265W	0.317	3.504E-02	TSC10
YOR289W	0.314	4.649E-02	-
YGR209C	0.312	1.424E-02	TRX2
YDL078C	0.308	1.365E-02	MDH3
YGR210C	0.298	3.941E-02	-
YGR019W	0.294	4.455E-02	UGA1
YJR117W	0.293	3.673E-02	STE24
YDL110C	0.288	4.055E-02	TMA17
YBR025C	0.286	1.921E-02	OLA1
YLR270W	0.279	4.956E-02	DCS1
YBR286W	0.276	2.857E-02	APE3

Supplementary Table 4 | Plasmids used in this study.

Plasmid name	Source	Identifier
pRS426	(Christianson et al., 1992)	N/A
pYES2-myc-20QmCh	(Park et al., 2013)	N/A
pYES2-myc-97QmCh	(Park et al., 2013)	N/A
pRS306P$_{GPD}$	(Mumberg et al., 1995)	N/A
pGEX-2T	GE Lifesciences	Cat. #28-9546-53
pFA6a-GFP(S65T)-KanMX6	(Bähler et al., 1998)	N/A
pRS306P$_{GPD}$GST-GFP-NLS	This study	pCLK207
pRS306P$_{GPD}$GST-mCh-NLS	This study	pCLK181
Ssa3-LacZ-Leu	(Liu et al., 1997)	N/A
pRS414P$_{GPD}$	(Mumberg et al., 1995)	N/A
pRS426Hsf1	(Holmes et al., 2014)	N/A
pRS414P$_{GPD}$Hsf1	This study	pCLK263
pRS425P$_{GAL}$	ATCC	Cat. #87331
pRS425P$_{GAL}$LacZ	This study	pCLK114
pESCLeu	Agilent	Cat. #217452
pESCLeu-20QmCh	This study	pCLK253
pESCLeu-97QmCh	This study	pCLK254
pRS313P$_{HSE}$LacZ	This study	pCLK270
Yeast ORF Collection	Dharmacon	Cat. #YSC3868
pRS414	(Sikorski and Hieter, 1989)	N/A
pRS414$_{GPD}$Sis1	(Douglas et al., 2008)	Addgene #18687
pRS414P$_{GPD}$Ydj1	This study	pCLK323
pRS414P$_{GPD}$DnaJb1	This study	pCLK210
pRS414P$_{GPD}$DnaJb6	This study	pCLK211
pYES2-Htt97Q-GFP	(Ripaud et al., 2014)	N/A
pESCUraRnqGFP	(Kaganovich et al., 2008)	N/A
P$_{UPR}$LacZ	(Cox et al., 1993)	N/A
pFA6a-KanMX4	(Wach et al., 1994)	N/A
pBacMam2-DiEx-LIC-C-flac_huntingtin_full-length_Q48	(Harding et al., 2019)	Addgene #111747
pYES2-myc-48QmCh	This study	pCLK334
pCM184	(Garí et al., 1997)	N/A
pCM184Sis1	This study	pCLK75
pRS414P$_{GPD}$HASis1	This study	pCLK331
pRS414P$_{GPD}$HAYdj1	This study	pCLK330
pRS414P$_{GAL}$HASis1	This study	pCLK301
pRS414P$_{GAL}$HASis1AAA	This study	pCLK302
pRS414P$_{GAL}$HASis1DG/F	This study	pCLK304
pRS414P$_{GAL}$HASis1DC	This study	pCLK313
pRS414P$_{GAL}$HASis1DDD	This study	pCLK315
pRS413P$_{GPD}$Ssa1	This study	pCLK242
pRS405P$_{ADH}$Ssa1GFP	This study	pCLK295
pRS413P$_{CYC}$	(Mumberg et al., 1995)	N/A
pRS413P$_{CYC}$Sis1	This study	pCLK292
pcDNA5/FRT/TO	Invitrogen	Cat. #V6520-20
FRT/TO V5 DnaJB6	(Hageman et al., 2010)	N/A
FRT/TO V5 DnaJB1	(Hageman et al., 2010)	N/A
FRT/TO V5 DnaJB6 H31Q	(Hageman et al., 2010)	N/A
FRT/TO V5 DnaJB6 M3	(Kakkar et al., 2016)	N/A
pCIneo-FlucDM	(Gupta et al., 2011)	N/A
mCherry-N1	Clontech	Cat. #632523
pYES2-FlucDM-mCh	This study	pCLK332

Supplementary Table 5│ Primers used in this study.

Primer name	Sequence
5 GST XbaI	AAAAtctagaatgtcccctatactagg
3 GST GS3 BHI	aaaaGGATCCggttctggttctggttcttttggaggatggtcgc
5 GFP BHI	AAAAggatccATGagtaaaggagaagaacttttc
3 GFP NLS SalI	AAAAgtcgacttaAACCTTTCTCTTCTTCTTTGGtttgtatagttcatccatg
5 mCh BHI	AAAAggatccATGGTGAGCAAGGGCG
3 mCh NLS SalI	AAAAgtcgacttaAACCTTTCTCTTCTTCTTTGGCTTGTACAGCTCG TCC
5 Hsf1 EcoRI	aaaaGAATTCATGAATAATGCTGCAAATAC
3 Hsf1 XhoI	aaaaCTCGAGCTATTTCTTAGCTCGTTTG
5 LacZ BamHI	aaaaGGATCCGGAGCTTGG
3 LacZ SalI	AAAAgtcgacTTATTTTTGACACCAGACC
5 pHSE BglII	AGCCagatctTTTAGGCTCGAAGATCC
5 Ydj1 SpeI	AAAAactagtATGGTTAAAGAAACTAAGTTT
3 Ydj1 BHI	AAAAggatccTCATTGAGATGCACATTG
5 V5 SpeI	AAAAactagtATGGGTAAGCCTATCCC
3 Dnajb1 XhoI	AAAActcgagctatattggaagaacctg
3 Dnajb6b XhoI	AAAActcgagttacttgttatccaagcg
PolyQBB_fw	GGCCCAGCTGTGGCTGAGGAG
Int_PolyQrev	GAAGCTTTTGAGGGACTCGAAG
5 Sis1 BamHI	aaaaGGATCCatggtcaaggagacaaaac
3 Sis1 NotI	aggcGCGGCCGCttaaaaattttcatctatagc
5 SpeI HASis1	aaaaACTAGTATGTCCTACCCAT
3 Sis1 BHI	aaaaGGATCCTTAAAAATTTTCATCTATAGC
5 SpeI HAYdj1	AAAAactagtATGTCCTACCCATACGATGTTCCAGATTACGCTGA TATGGTTAAAGAAACTAAGTT
3 Ydj1 BHI	AAAAggatccTCATTGAGATGCACATTG
3 Sis1-121 BHI	aaaaGGATCCttaGCCGCCAAAGAATTGTG
3 Sis1-338 BHI	AAAAggatccttaTATTGGATAGTCCACTTTA
pRS_Ssa1_fw	TAGAACTAGTGGATCCCCCGGGCTGCAGGAATGTCAAAAGCT GTCGGTATTG
Ssa1_GS_rev	TTCTCCTTTACTGGAGCCACTACCGGAACCATCAACTTCTTCA ACGGTTGG
GS_GFP_fw	GGTTCCGGTAGTGGCTCCAGTAAAGGAGAAGAACTTTTCACTG
GFP_pRS_rev	TCGACGGTATCGATAAGCTTGATATCGAATCTATTTGTATAGTT CATCCATGCCATG
pRS_MCS_fw	ATTCGATATCAAGCTTATCGATACCGTC
pRS_MCS_rev	TCCTGCAGCCCGGGGG
5 Sis1-GFP	ACTAAACGACGCTCAAAAACGTGCTATAGATGAAAATTT TGGTTCTGGTTCTGGTTCTCGGATCCCCGGGTTAATTAA
3 Sis1-GFP	ATTTATTTGAGTTTATAATTATATTTGCTTAGGATTACTAG AATTCGAGCTCGTTTAAAC

Supplementary Table 6 │ Experimental models: cell lines and yeast strains used in this study.

Model	Name	Source	Identifier
S. cerevisiae	YPH499: *MAT*a *ura3-52 lys2-801_amber ade2-101_ochre trp1-Δ63 his3-Δ200 leu2-Δ1*	(Sikorski and Hieter, 1989)	N/A
S. cerevisiae	YPH499 *ura3-52::URA::*P_{GPD}*-GST-GFP-NLS*	This study	yCLK225
S. cerevisiae	YPH499 *leu2-Δ1::LEU::*P_{ADH}*-SSA1-GFP*	This study	yCLK250
S. cerevisiae	YPH499 *Δssa1::KANMX4*	This study	yCLK243
S. cerevisiae	YPH499 *SIS1GFP::KANMX6*	This study	yCLK207
S. cerevisiae	YPH499 *leu2-Δ1::LEU::*P_{ADH}*-SSA1-GFP ura3-52::URA::*P_{GPD}*-GST-mCh-NLS*	This study	yCLK255
S. cerevisiae	YPH499 *SIS1GFP::KANMX6 ura3-52::URA::*P_{GPD}*-GST-mCh-NLS*	This study	yCLK256
Human cell line	HEK293T	ATCC	ATCC CRL-3216
Human cell line	HEK293T DNAJB6$^{-/-}$	(Thiruvalluvan et al., 2020)	N/A

Supplementary Table 7│ Antibodies used in this study.

Antibody	Dilution	Source	Catalog No.	RRID
mCherry monoclonal antibody (rat)	1:1,000	Invitrogen	M11217	AB_2536611
Anti-Hsp104 polyclonal antibody (rabbit)	1:1,000	Abcam	ab2924	AB_2041710
Hsp26 Antibody (rabbit)	1:5,000	Johannes Buchner (Technische Universität München)	N/A	N/A
PGK1 monoclonal antibody (mouse)	1:1,000	Invitrogen	459250	AB_2532235
Sis1 Antibody (rabbit)	1:10,000	Cosmo Bio	COP-080051	AB_10709957
Ydj1 Antibody (rabbit)	1:10,000	Elizabeth Craig (University of Wisconsin-Madison)	N/A	N/A
Anti-HA High Affinity (rat)	1:1,000	Roche	11867423001	AB_390918
Anti-v5 tag monoclonal antibody (mouse)	1:1,000	Abcam	ab27671	AB_471093
Ssa1/2 Antibody (rabbit)	1:10,000	Elizabeth Craig (University of Wisconsin-Madison)	N/A	N/A
HSP70/HSP72 monoclonal antibody (mouse)	1:1,000	Enzo Life Sciences	ADI-SPA-810	AB_10616513
HSP70B monoclonal antibody (mouse)	1:2,000	Enzo Life Sciences	ADI-SPA-754	AB_10615942
HSP27 polyclonal antibody (rabbit)	1:1,000	Enzo Life Sciences	ADI-SPA-803	AB_10615084
GAPDH monoclonal antibody (mouse)	1:10,000	Fitzgerald	10R-G109A	AB_1285808
Andi-DnaJB6 (rabbit)	1:2,000	Ineke Braakman (Utrecht University)	N/A	N/A
Recombinant Anti-Hsf1 antibody (rabbit)	1:30,000	Abcam	ab52757	AB_880518
Anti-HSF1 (phosphor S326) antibody (rabbit)	1:5,000	Abcam	ab76076	AB_1310328
V5 Tag monoclonal antibody (mouse)	1:5,000	Invitrogen	46-0705	AB_2556564
