# Supplementary material for: Sis1 potentiates the stress response to protein aggregation and elevated temperature
Source: Nat Commun. 2020 Dec 8;11:6271. doi: 10.1038/s41467-020-20000-x (PMC7722728; doi:10.1038/s41467-020-20000-x)
Supplement: Supplementary file 3 — Reporting summary [file 41467_2020_20000_MOESM3_ESM.pdf]

nature research

Corresponding author(s): F. Ulrich Hartl

Last updated by author(s): Oct 26, 2020

Reporting Summary

Nature Research wishes to improve the reproducibility of the work that we publish. This form provides structure for consistency and transparency in reporting. For further information on Nature Research policies, see our Editorial Policies and the Editorial Policy Checklist.

Statistics

For all statistical analyses, confirm that the following items are present in the figure legend, table legend, main text, or Methods section.

n/a	Confirmed	
☐	☒	The exact sample size (n) for each experimental group/condition, given as a discrete number and unit of measurement
☐	☒	A statement on whether measurements were taken from distinct samples or whether the same sample was measured repeatedly
☐	☒	The statistical test(s) used AND whether they are one- or two-sided<br>*Only common tests should be described solely by name; describe more complex techniques in the Methods section.*
☒	☐	A description of all covariates tested
☐	☒	A description of any assumptions or corrections, such as tests of normality and adjustment for multiple comparisons
☐	☒	A full description of the statistical parameters including central tendency (e.g. means) or other basic estimates (e.g. regression coefficient) AND variation (e.g. standard deviation) or associated estimates of uncertainty (e.g. confidence intervals)
☐	☒	For null hypothesis testing, the test statistic (e.g. F, t, r) with confidence intervals, effect sizes, degrees of freedom and P value noted<br>*Give P values as exact values whenever suitable.*
☒	☐	For Bayesian analysis, information on the choice of priors and Markov chain Monte Carlo settings
☒	☐	For hierarchical and complex designs, identification of the appropriate level for tests and full reporting of outcomes
☒	☐	Estimates of effect sizes (e.g. Cohen's d, Pearson's r), indicating how they were calculated

Our web collection on statistics for biologists contains articles on many of the points above.

Software and code

Policy information about availability of computer code

Data collection	No novel software was used for data collection.
Data analysis	LI-COR Image Studio Lite (v 5.2.5, https://www.licor.com/bio/image-studio-lite/download), Bio-Rad Image Lab Software (v 6.0.1, https://www.bio-rad.com), String Database (v 11, Szklarczyk et al., 2019; https://string-db.org), Fiji (v 2, Schindelin et al., 2012; https://imagej.net/Fiji/Downloads), GraphPad QuickCalcs (https://www.graphpad.com/quickcalcs/ttest1.cfm), GraphPad Prism (v 8, https://www.graphpad.com/scientific-software/prism/). DEseq R package (Anders and Huber, 2010, https://bioconductor.riken.jp/packages/3.0/bioc/html/DESeq.html). GOseq R package (Young et. al, 2010; http://bioconductor.org/packages/release/bioc/html/goseq.html).

For manuscripts utilizing custom algorithms or software that are central to the research but not yet described in published literature, software must be made available to editors and reviewers. We strongly encourage code deposition in a community repository (e.g. GitHub). See the Nature Research guidelines for submitting code & software for further information.

Data

Policy information about availability of data

All manuscripts must include a data availability statement. This statement should provide the following information, where applicable:
- Accession codes, unique identifiers, or web links for publicly available datasets
- A list of figures that have associated raw data
- A description of any restrictions on data availability

The transcriptome data discussed in this publication have been deposited in NCBI's gene expression omnibus and are accessible through GEO series accession number GSE151215 (https://www.ncbi.nlm.nih.gov/geo/query/acc.cgi?acc=GSE151215). The string database is available at https://string-db.org (v 11, organism S. cerevisiae). All other data supporting the findings of this study are available within the paper or accompanying source data file.

Field-specific reporting

Please select the one below that is the best fit for your research. If you are not sure, read the appropriate sections before making your selection.

- [x] Life sciences
- [ ] Behavioural & social sciences
- [ ] Ecological, evolutionary & environmental sciences

For a reference copy of the document with all sections, see nature.com/documents/nr-reporting-summary-flat.pdf

Life sciences study design

All studies must disclose on these points even when the disclosure is negative.

Sample size	No statistical methods were used to predetermine sample size. Biochemical experiments were performed in triplicate on biologically independent samples as per commonly accepted field standards and to enable statistical analysis (e.g. calculation of mean and SD).
Data exclusions	No data were excluded.
Replication	All experiments were replicated at least three times. Attempts at replication were successful.
Randomization	Not applicable. Experiments were performed comparing comparing strains/lines that were identical except for the specific experimental treatment tested.
Blinding	Not applicable. Experiments were performed comparing various treatments on otherwise comparable samples and as such it was necessary for the researchers to be aware of the treatment applied (e.g. transformation/heat exposure/etc.). Appropriate cellular and biochemical controls were included in each experimental replication and results reported are predominantly quantitative in nature.

Reporting for specific materials, systems and methods

We require information from authors about some types of materials, experimental systems and methods used in many studies. Here, indicate whether each material, system or method listed is relevant to your study. If you are not sure if a list item applies to your research, read the appropriate section before selecting a response.

Materials & experimental systems

n/a	Involved in the study	
[ ]	[x]	Antibodies
[ ]	[x]	Eukaryotic cell lines
[x]	[ ]	Palaeontology and archaeology
[x]	[ ]	Animals and other organisms
[x]	[ ]	Human research participants
[x]	[ ]	Clinical data
[x]	[ ]	Dual use research of concern

Methods

n/a	Involved in the study	
[x]	[ ]	ChIP-seq
[x]	[ ]	Flow cytometry
[x]	[ ]	MRI-based neuroimaging

Antibodies

Antibodies used	mCherry monoclonal (Cat#M11217, RRID: AB_2536611), PGK1 monoclonal (Cat#459250, RRID:AB_2532235) and V5 monoclonal (Cat#46-0705, RRID:AB_2556564) were obtained from Invitrogen. Hsp104 polyclonal (Cat#ab2924, RRID:AB_2041710), V5 monoclonal (Cat#ab27671, RRID:AB_471093), Hsf1 (Cat#ab52757, RRID:AB_880518), and Hsf1 Phosphor S326 (Cat#ab76076, RRID:AB_1310328) were obtained from Abcam. Hsp70/72 monoclonal (Cat#ADI-SPA-810, RRID:AB_10616513), Hsp70B monoclonal (Cat#ADI-SPA-754, RRID:AB_10615942) and Hsp27 polyclonal (Cat#ADI-SPA-803, RRID:AB_10615084) were obtained from Enzo Life Sciences. Sis1 was obtained from Cosmo Bio (Cat#COP-080051, RRID:AB_10709957). HA high affinity was obtained from Roche (Cat#11867423001, RRID:AB_390918). GAPDH monoclonal was obtained from Fitzgerald (Cat#10R-G109A, RRID:AB_880518). Hsp26 antibody was provided by Johannes Buchner (Technische Universitaet Muenchen), Ssa1/2 and Ydj1 antibodies were provided by Elizabeth Craig (University of Wisconsin-Madison) and DnaJB6 antibody was provided by Ineke Braakman (Utrecht University). Anti-Rat (Cat#A9037, RRID:AB_258429), Anti-Rabbit (Cat#9169, RRID:AB_258434, and Anti-Mouse (Cat#A4416, RRID:AB_258167) IgG (whole molecule)-peroxidase secondary antibodies produced in goat were obtained from Sigma-Aldrich.
Validation	All antibodies have been previously used for the application and species described here. mCherry has been validated for Western blotting of recombinant protein (https://assets.thermofisher.com/TFS-Assets/LSG/manuals/mCherry_rat_monoclonal_ab_man.pdf), PGK has been validated for Western blotting in yeast (https://www.thermofisher.com/order/genome-database/dataSheetPdf?producttype=antibody&productsubtype=antibody_primary&productId=459250&version=123), V5 has been validated for Western blotting of recombinant protein (https://www.thermofisher.com/order/genome-database/dataSheetPdf?producttype=antibody&productsubtype=antibody_primary&productId=R960-25&version=123 and https://www.abcam.com/v5-tag-antibody-sv5-pk1-ab27671.html), Hsp104 has been validated for Western blotting in yeast (https://www.abcam.com/hsp104-antibody-ab2924.html), Hsf1 has been validated for Western blotting in human cells (https://www.abcam.com/hsf1-antibody-ep1710y-chip-grade-ab52757.html), Phospho-Hsf1 has been validated for Western blotting in human cells (https://www.abcam.com/

hsf1-phospho-s326-antibody-ep1713y-ab76076.html), Hsp70/72 has been validated for Western blotting in human cells (https://www.enzolifesciences.com/ADI-SPA-810/hsp70-hsp72-monoclonal-antibody-c92f3a-5/), Hsp70B has been validated for Western blotting in human cells (https://www.enzolifesciences.com/ADI-SPA-754/hsp70b-monoclonal-antibody-165f/), Hsp27 has been validated for Western blotting in human cells (https://www.enzolifesciences.com/ADI-SPA-803/hsp27-polyclonal-antibody/), Sis1 has been validated for Western blotting in yeast (https://search.cosmobio.co.jp/cosmo_search_p/search_gate2/docs/COP_/COP080051.20100628.pdf), HA has been validated for Western blotting of recombinant protein (https://www.sigmaaldrich.com/catalog/product/roche/roahaha?lang=de&region=DE), GAPDH has been validated for Western blotting of human protein (https://www.fitzgerald-fii.com/gapdh-antibody-10r-g109a.html).

Eukaryotic cell lines

Policy information about cell lines

Cell line source(s)	HEK293T cells: ATCC CRL-3216; HEK293T DNAJB6-/- cells: Harm Kampinga Lab, University Medical Center Groningen, described in Thiruvalluvan et al., Mol Cell, 2020.
Authentication	Commercial HEK293T cell lines were not personally authenticated. The DNAJB6-/- cell line was confirmed by immunoblotting for DNAJB6.
Mycoplasma contamination	Cell lines were not personally tested for mycoplasma contamination.
Commonly misidentified lines (See ICLAC register)	No commonly misidentified lines were used in this study.
